# Neuroprotective Effect Against Ischemic Stroke of the Novel Functional Drink Containing Anthocyanin and Dietary Fiber Enriched-Functional Ingredient from the Mixture of Banana and Germinated Jasmine Rice

**DOI:** 10.3390/life15081222

**Published:** 2025-08-02

**Authors:** Mubarak Muhammad, Jintanaporn Wattanathorn, Wipawee Thukham-mee, Sophida Phuthong, Supaporn Muchimapura

**Affiliations:** 1 Neuroscience Program, Department of Physiology, Graduate School, Faculty of Medicine, Khon Kaen University, Khon Kaen 40002, Thailand; mubarak.m@kkumail.com; 2 Department of Physiology, Faculty of Medicine, Khon Kaen University, Khon Kaen 40002, Thailand; meewep@gmail.com (W.T.-m.); sophiphu@kku.ac.th (S.P.); supmuc@kku.ac.th (S.M.); 3 Research Institute for High Human Performance and Health Promotion, Khon Kaen University, Khon Kaen 40002, Thailand

**Keywords:** ischemic stroke, anthocyanin, dietary fiber, banana, black Jasmine rice

## Abstract

Due to the stroke-protective effects of dietary fiber and anthocyanin together with the synergistic interaction, we hypothesized that the functional drink containing the anthocyanins and dietary fiber-enriched functional ingredient from banana and germinated black Jasmine rice (BR) should protect against ischemic stroke. BR at doses of 300, 600, and 900 mg/kg body weight (BW) was orally given to male Wistar rats weighing 290–350 g once daily for 21 days, and they were subjected to ischemic reperfusion injury induced by temporary occlusion of the middle cerebral artery (MCAO/IR) for 90 min. The treatment was prolonged for 21 days after MCAO/IR. They were assessed for brain infarction volume, neuron density, Nrf2, MDA, and catalase in the cortex together with serum TNF-α and IL-6. *Lactobacillus* and *Bifidobacterium* spp. in feces were also assessed. Our results showed that BR improved the increase in brain infarcted volume, MDA, TNF-α, and IL-6 and the decrease in neuron density, Nrf2, catalase, and both bacteria spp. induced by MCAO/IR. These data suggest the stroke-protective effect of the novel functional drink, and the action may involve the improvement of Nrf2, oxidative stress, inflammation, and the amount of *Lactobacillus* and *Bifidobacterium* spp.

## 1. Introduction

Stroke, the second leading global cause of death, is the most common cause of permanent disability [1]. It has been reported that over 62% of global stroke cases are ischemic strokes [2]. It has been shown that the currently accepted therapies are the application of the intravenous recombinant thrombolytic agent tissue plasminogen activator (tPA) and endovascular thrombectomy with stent-retriever devices combined with advanced imaging modalities. Unfortunately, an excellent functional outcome induced by tPA appears to have a limited therapeutic time window and the potential side effect of intracranial hemorrhage [3], whereas the limitation of endovascular thrombectomy appears to depend on personal experience and skill [4]. In addition, over 80% of stroke survivors experience sensorimotor and cognitive impairments [5,6], which in turn impair patients’ quality of life [7]. Based on the multi-aspect impairment mentioned earlier, the multidisciplinary team and the advanced technology-based are required [8]. Owing to the challenges in rehabilitation difficulty [9] and the limitation of the current therapeutic efficacy [10,11], stroke prevention strategy has gained much attention.

A pile of evidence has demonstrated that oxidative stress and inflammation are closely intertwined with each other, and they play important roles in the development and progress of ischemic stroke [12]. Oxidative stress can induce injury to endothelial cells and platelets, leading to thrombus formation, maturation, and stability [13], whereas inflammation modulates the thrombo-inflammatory cascade pathway by inducing an accumulation of inflammatory cells at the site of endothelial injury and by stimulating platelets, which in turn induces and modulates the clotting process and activates thrombus formation [14,15]. Owing to the crucial roles of oxidative stress and inflammation in stroke development and progression of stroke mentioned earlier, they have been focused on as the targets of protection and treatment strategies.

Currently, the concept of an intervention of food as medicine is regarded as an emerging trend of preventive medicine [16]. Diet quality has been focused on as a primary intervention for stroke prevention. A pile of evidence in this decade reveals that a Mediterranean diet [17] and polyphenol-enriched substances such as fruits and vegetables show an inverse relationship with stroke risk [18]. Antioxidants such as vitamin C and vitamin E also decrease the risk of ischemic stroke [19]. In addition to antioxidants, dietary fiber also decreases the risk of stroke. Different types of dietary fiber appear to show different effects on stroke. Total fiber, insoluble fiber, soluble fiber, fruit fiber, and vegetable fiber produce a significant reduction in stroke risk, whereas cereal fiber fails to produce a significant reduction in this parameter [20]. Recently, it has been demonstrated that a single-agent therapy is difficult to achieve the ultimate goal of stroke prevention due to the dose-limiting toxicity. Therefore, an increase in the efficacy of the prevention strategy may be raised by the combination effect of antioxidants and dietary fiber.

Black Jasmin rice, or *Oryza sativa* L., a plant in the family of Poaceae, contains high anthocyanin and dietary fiber content [21], exhibits both antioxidant and anti-inflammatory effects, and provides health benefits for brain and cardiovascular functions [22]. The beneficial effects of germinated black rice also provided higher benefits than normal black rice [23]. Beyond the black Jasmine rice, banana, or *Musa sapientum* var. Nam Wa, also provides brain health benefits. Both the pulp and peel of a banana exert anti-anxiety and antidepressant effects. Furthermore, they also strengthen memory [24]. Banana pulp is a rich source of amine compounds, phenolic compounds, and vitamins (B3, B6, B12, C, and E) together with dietary fiber [25], whereas banana peel is abundant with dietary fiber, crude protein, carbohydrates (cellulose, hemicellulose, pectin, and lignin), minerals (calcium, magnesium, phosphorus, and potassium), amino acids (leucine, threonine, valine, and phenylalanine), and polyunsaturated fatty acids, particularly linoleic acid [26]. Both the pulp and peel of bananas also possess antioxidant and anti-inflammatory properties [27,28].

Owing to the antioxidant and anti-inflammatory effects of germinated black rice and banana together with the synergistic interaction mentioned earlier, we hypothesized that an anthocyanin- and dietary fiber-enriched functional drink from banana and black Jasmine rice could optimize the protective effect against ischemic stroke by acting via multi-pathway. To the best of our knowledge, no scientific evidence regarding this issue has been available until now. Therefore, we aimed to determine the neuroprotective effect against ischemic stroke in an animal model induced by an occlusion of the right middle cerebral artery of the novel functional drink. Furthermore, the possible underlying mechanisms were also explored.

## 2. Materials and Methods

### 2.1. Preparation of an Anthocyanin and Dietary Fiber-Enriched Functional Drink from Banana and Black Jasmine Rice

Thai black rice (variety Thai Jasmine Black Rice) and banana (Kluai Khai harvested from October 2022 to December 2022) were obtained from Maha Sarakham province, northeastern Thailand, Thailand. They were authenticated by a taxonomist, Nattawut Triyuthchai, at the Khon Kaen University (KKU) plant museum, and a sample was also deposited at the Research Institute for Human High Performance and Health Promotion, Khon Kaen University, Khon Kaen, Thailand. The assigned authentication numbers include KKU-No 25552 for Thai black rice and KKU-No 25551 for Kluai Khai.

Banana, or *Musa sapientum* (var. Kluai Khai), at a 5-day ripening period was washed and separated into peel and pulp. The fresh banana pulp was immediately weighed and kept at −20 °C until used for the preparation of banana puree, while the peel part was thoroughly washed with water containing salt, cut into pieces, weighed, and dried in the oven (Memert GmbH, Eagle, WI, USA) at 50 °C for 12 h. The dried peel was weighed and soaked with 95% hydroalcoholic solution for 24 h. The resultant peel was dried in an incubator for 12 h and blended to obtain the banana peel-derived dietary fiber powder.

Rice, or *Oryza sativa* L. Indica (var. black Jasmine rice), was used as raw material for the preparation of germinated black Jasmine rice extract. Firstly, the impurities were removed, then it was weighed. Then, it was washed two times and soaked in water for 24 h. The soaked product was then wrapped with a sterilized white cloth before incubating in a box for 12 h. The germinated rice was then collected, dried for 12 h in an incubator, blended, and subsequently prepared as a 95% hydroalcoholic extract by using a maceration technique.

**Figure 1:**
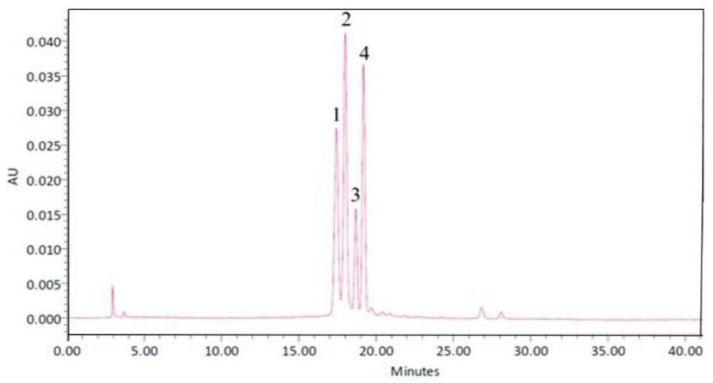
Profile of the anthocyanin- and dietary fiber-enriched functional drink from banana and black jasmine rice at a concentration of 500 mg/mL determined by high-performance liquid chromatography (HPLC) at the wavelength of 370 nm. The number 1 represented delphinidin 3-O-glucoside, number 2 represented delphinidin-3-O-rutinoside, number 3 represented cyanidin-3-O-glucoside, and 4 represented cyanidin-3-O-rutinoside.

The mixture of banana pulp puree was mixed with a germinated black Jasmine rice extract at a ratio of 30:70 because our in vitro data showed that this ratio revealed the highest biological activities related to the pathophysiology of stroke, including antioxidant and anti-inflammatory activities. Then, the obtained mixture was mixed with 1 g of banana peel-derived dietary fiber and served as the functional ingredient. To prepare the functional drink, the developed functional ingredient was mixed with germinated black Jasmine rice milk at the concentrations of 0.725%, 0.950%, and 1.175%, respectively, while the placebo was prepared in a similar pattern without the functional ingredient derived from banana and germinated black Jasmine rice. The main ingredient of the developed functional drink is anthocyanin, as shown in Figure 1.

### 2.2. Study Design

All animals used in this part were male adult Wistar rats (20-week-old) weighing 290–350 g from Nomura Siam International Co., Ltd. (Bangkok, Thailand). They were maintained in standard condition (23 ± 2 °C, 12:12 h light–dark cycle), and bedding was changed every 3 days. The unlimited access to Perfect Companion Rat Food No. 082 (Perfect Companion Group Co., Ltd., Bang Sao Thong, Samit Prakarn, Thailand) and water was provided. The authentication approval of the experimental protocol was performed by the Institutional Animal Care and Use Committee of Khon Kaen University (Record number IACUC-KKU-16/66, approval date 16 February 2023). After a 1-week acclimatization period, they were randomly assigned into 10 experimental groups of 6 rats each as follows:

Group I (Naïve Control): all rats in this group received no treatment and served as the negative control group.
Group II (Sham + vehicle): the rats in this group were orally given placebo treatment and subjected to sham operation. This group was designed to determine the effect of the operation procedures and placebo on the observed parameters, particularly brain damage and dysfunction, in this study.
Group III (MCAO + Vehicle): the experimental rats received a placebo and were subjected to a temporary occlusion of the right middle cerebral artery for 90 min (MCAO/IR). This group provided the information regarding the effect of MCAO/IR and placebo on the observed parameters, particularly brain damage and dysfunction, in this study.
Group IV (Banana peel-derived dietary fiber 200 + MCAO): the animals in this group were orally administered banana peel-derived dietary fiber at the same dose as that presented in the functional drink at a dose of 200 mg/kg body weight (BW) and received MCAO/IR. This group was designed to provide information about the effect of banana peel-derived dietary fiber alone on the observed parameters, particularly brain damage and dysfunction in this study.
Group V (Banana puree 200 + MCAO): all rats in this group were orally administered banana pulp puree at the same dose as that presented in the functional drink at a dose of 200 mg/kg BW and received MCAO/IR. Information obtained from this group emphasized the effect of banana puree alone on the observed parameters. particularly brain damage and dysfunction in this study.
Group VI (Rice 200 + MCAO): the experimental rats in this group received germinated black Jasmine rice extract at the same dose as that presented in the functional drink at a dose of 200 mg/kg BW and were subjected to MCAO/IR. The effects of germinated black Jasmine rice extract alone on the observed parameters, particularly brain damage and dysfunction, were obtained from this group.
Group VII (Piracetam 200 + MCAO): this group served as the positive control group. All experimental animals were orally administered piracetam at a dose of 200 mg/kg BW and subjected to MCAO/IR. This group served as the positive control or was treated with a standard drug targeting cerebral blood flow enhancement [29].
Group VIII–X (BR + MCAO): rats in groups VIII, IX, and X were orally administered the functional drink containing the functional ingredient from banana and black jasmine rice (BR) at the doses of 300, 600, and 900 ma/kg BW, respectively. These doses were selected based on the transference of in vitro data regarding antioxidant activity to the in vivo experiment. Following the treatment, they were subjected to MCAO/IR. These groups were experimental groups.

**Figure 2:**
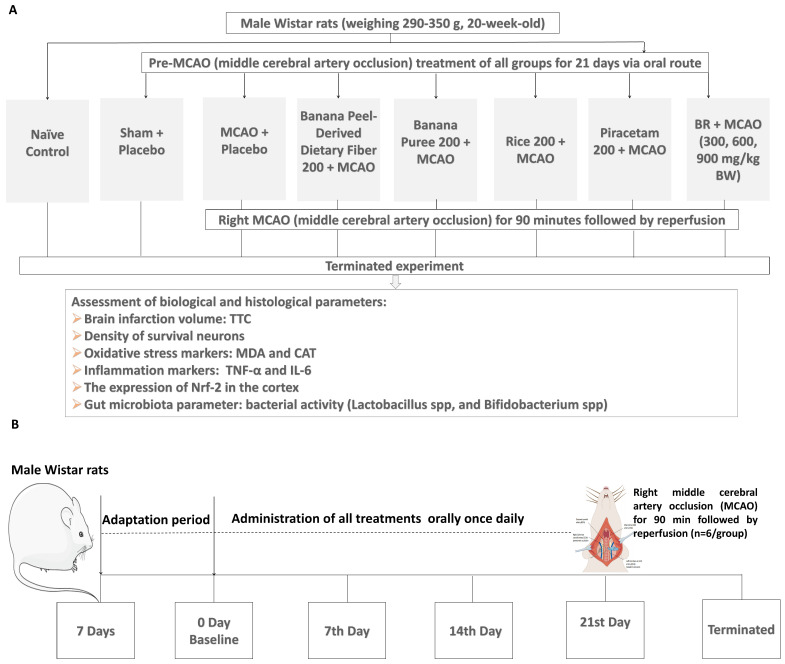
The summary diagram demonstrates study design. (**A**) Schematic diagram showing the experimental groups, the treatments administered, and the measured parameters assessed in this study. (**B**) The time window of treatment administration.

In this part, all substance administrations were performed once daily for a period of 21 days prior to the induction of MCAO/IR as illustrated in Figure 2 study design. To avoid the confounding errors, we used 3 sets of animals. The first set was used for the determination of brain infarcted volume, the second set was used for the assessment of neuron density in the cerebral cortex, and the last set was used for the measurements of all changes in biomarkers, including oxidative stress, inflammation, and the expression of nuclear factor erythroid 2-related factor 2 (Nrf2).

### 2.3. The Temporary Occlusion of Middle Cerebral Artery Induced by Ischemia-Reperfusion (MCAO/IR)

Rats were fasted overnight and subjected to anesthetization with pentobarbital sodium at doses of 50 mg/kg BW via the intraperitoneal route. An absence of withdrawal reflex should be detected before an induction of midline skin incision at the neck to expose the right common carotid artery (CCA). Following this step, a silicone-coated 4-0 monofilament nylon was gently introduced and inserted from the lumen of the right common carotid artery (CCA) down through the internal carotid artery (ICA) around 17–18 mm from the bifurcation. Then, a 90-min occlusion of the right MCAO, reperfusion injury, was performed by an abrupt withdrawal of the nylon monofilament. The body temperature of the rats was maintained at 37 °C throughout the procedure, and the rats were kept under care from secretions by regular suctioning.

### 2.4. Brain Water Content Assessment

Brain water content was calculated from the equation below [30], for which body weight was assessed and recorded at baseline and every week until the 21st day. The changes in body weight weekly were analyzed, and the termination day body weight was used together with the brain weight separated after termination to compute the percentage brain water content as follows:

Brain water content (%) = (brain weight/body weight) × 100%

### 2.5. Neuronal Density Assessment

Histological technique was employed to determine the cortex’s surviving neuronal density. In brief, the brain was fixed in 4% paraformaldehyde at a pH of 7.4 and a temperature of 4 °C. The integrity of neuronal tissue architecture was ensured by introducing the brain into the 30% formalin sucrose solution for 2–3 days, prior to which the cortex cryostat sections were prepared by cutting at 10 μm thickness. Sections were attached to slides covered with 0.3% gelatine buffer and 0.05% aluminum potassium sulfate. For staining, each section was first dehydrated in descending grades of ethanol. Following this step, each section was rehydrated in various grades of xylene. Slides were then immersed in crystal violet staining solution for 8–10 min, followed by decolorization in acetic acid, dehydration, and cover slipping with Permount. They were then finally mounted and microscopically analyzed for neuronal density in Layer III (External pyramidal layer) and Layer V (Internal pyramidal layer). Each slide was analyzed for 50 fields. Data were presented as number of cells/255 μm^2^ [31].

### 2.6. Determination of Oxidative Stress Markers

The oxidative stress markers such as malondialdehyde (MDA) level and catalase (CAT) activity were explored in this study according to the method of Wattanathorn et al. [32]. MDA level was determined by using the thiobarbituric (TBA) reactive substances method. Various solutions such as 10% trichloroacetic acid (TCA: Merck Chemical, Darmstadt, Germany), 0.6% thiobarbituric acid (TBA: Sigma-Aldrich, Saint Louis, MO, USA), 8% sodium dodecyl sulfate (SDS: Bio Basic Inc., Markham, ON, Canada), 0.5% BHT (2,6 Di-tert-butyl-4-methylphenol), and 5.5 mM ethylenediaminetetraacetic acid (EDTA: Sigma-Aldrich, Saint Louis, MO, USA) were freshly prepared. A combined mixture containing 150 μL of brain homogenate, 125 μL each of TCA, EDTA, and SDS, and 10 μL of BHT was vortexed and incubated at room temperature for 10 min. A 535 μL of TBA was added to the resultant mixture, then boiled for 30 min at 100 °C. Then the solution was allowed to cool down for 3 min and centrifuged for 10 min at 10,000 rpm. The supernatant was collected and determined to have an absorbance of 532 nm. Triethyl phosphate (TEP) at the concentrations between 0.330 and 9.888 μmol/L was prepared as a standard calibration curve. The brain tissue MDA marker was expressed as μmol/L protein.

For the catalase assessment, various solutions such as 0.065 M hydrogen peroxide (H_2_O_2_), 60 mmol/L sodium-potassium phosphate buffer (pH 7.4), and 32.4 mmol/L ammonium molybdate (Kemaus, Cherrybrook, N.S.W., Australia) were prepared. The tissue sample was diluted with buffer in a 5× ratio, and 20 μL of the sample was combined with 100 μL of substrate (H_2_O_2_), then mixed by vortex and incubated at 37 °C for 2 min. A 100 μL of ammonium molybdate was subsequently included, and the absorption was read at 405 nm against the blank containing 120 μL of phosphate buffer and 100 μL of ammonium molybdate. Bovine liver catalase (Sigma-Aldrich, Saint Louis, MO, USA) at the concentrations between 1.5625 and 100 U/mL was prepared as a standard curve. Catalase activity was calculated and expressed as kU/L.

### 2.7. Assessment of Inflammatory and Immune Markers in Serum

Elisa kits for TNF-α (ab108913, Abcam, Waltham, MA, USA) and IL-6 (catalogue number EZMIL6, Millipore, MA, USA) were performed according to the manufacturer’s manual guidance. For TNF-α assessment, 50 μL of the samples were added per well, followed by 50 μL of each substance, including 1× biotinylated TNF-alpha antibody, 1× SP conjugate, chromogen substrate, and stop solution (which changed the color of the reaction from blue to yellow), while washing five times between each step with 1× wash buffer. An absorbance at 450 nm was measured by using the microplate reader.

For the IL-6 assay, 50 μL of the samples were added to each well, followed by the addition of 100 μL of the following substances, including IL-6 detection antibody, Avidin-HRP, substrate solution F, and stop solution (termination reaction step by changing solution color from blue to yellow). An absorbance at 450 nm was detected. In all cases, the standards are treated as the sample together using the same procedure. The optical density (OD) of the standards was obtained, and the calibration curve was plotted using the standard concentration and their obtained OD. The results for all the assay concentrations in the sample were finally calculated from the equation of the curve, respectively.

### 2.8. Assessment of Nrf2 Expression in Cortex

Assessment of Nrf2 was carried out using western blot assessment. Brain was homogenized with an extraction solution cell lysis buffer (10× #9803S). The homogenized samples were centrifuged at 14,000× *g* at 4 °C for 30 min. Then, an aliquot of tissue samples at a volume of 25 μL was loaded onto an SDS-polyacrylamide gel, and the protein was separated by SDS-PAGE gel electrophoresis. After an electrophoresis process, protein bands were transferred from the gel and were transferred to a nitrocellulose membrane. Then, the membrane was washed with 1× TBS-T and incubated in a blocking solution of 5% BSA (bovine serum albumin) at room temperature for 1 h while shaking to block the reaction process. Then, the membrane was incubated with the dilution of Nrf2, a primary antibody (1:500 μL), at 4 °C overnight while shaking. At the end of an incubation, the membrane was washed with 1× T-PBS for 30 min. Then, it was incubated with a secondary antibody (antirabbit IgG), which is conjugated with an enzyme, at room temperature for 1 h. Then, the membrane was washed to remove unbound antibody. An evaluation of the protein band densities was performed with the ECL system and the luminescent image analyzer (LAS-4000, GE Healthcare, Marlborough, MA, USA), and the ImageJ (version 1.5.4) software system.

### 2.9. Determination of Lactobacillus and Bifidobacterium in the Fecal Samples

The fecal samples collected at baseline (pre-MCAO) and after MCAO (postMCAO) were diluted with phosphate buffer (pH 7.4) (1:9 *w*/*v*) and prepared as a serial dilution at the concentration range between 10^−2^ and 10^−8^ by using the same diluent. Then, a 100 μL aliquot of each concentration was inoculated in duplicate by surface spreading on De Man–Rogosa–Sharpe (MRS) agar plates (Himedia™ Lactobacillus MRS agar and lactic acid-producing bacteria) and Bifidobacterium agar (Himedia™). Then, they were incubated at 37 °C for 48 h in a W-Zip standing Pouch by placing GasPak™ anaerobic (MGC, Mitsubishi, Chiyoda-ku, Japan) into the W-Zip bag to induce anaerobic conditions. At the end of an incubation, all plates were removed, and lactic acid-producing bacteria, *Lactobacillus* spp., and *Bifidobacterium* spp. were separately isolated and counted according to the different morphology on culture plates and Gram stain under microscopes. Data were expressed as log Colony Forming Unit (CFU)/mL.

### 2.10. Statistical Analysis

All data are presented as the mean ± S.E.M. (standard error of mean). One-way ANOVA (analysis of variance) followed by a Post Hoc LSD (least significant difference) statistical test was employed to test the statistical difference between variables. The statistical analysis was executed using computerized SPSS (statistical package for the social sciences) version 21 for Windows. All statistical differences between variables were considered significant when the *p*-value was < 0.05.

## 3. Results

### 3.1. Changes in Brain Infarcted Volume

**Figure 3:**
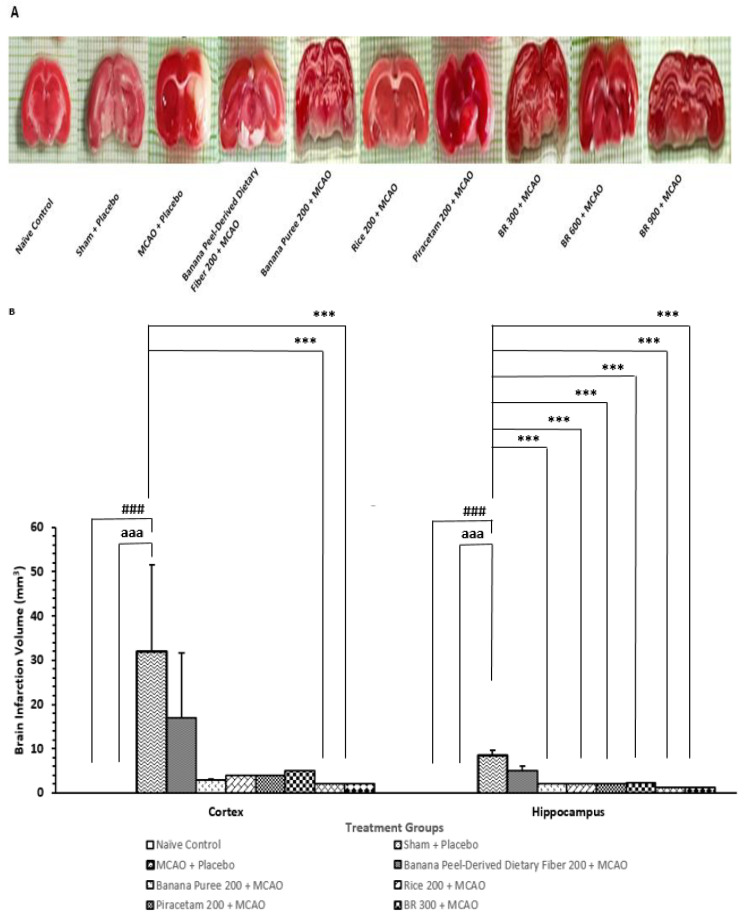
Changes in brain infarcted volume of various treatment groups. (**A**) Representative picture of brain infarcted volume assessed 24 h after reperfusion. (**B**) Bar graph showing quantitative data of infarcted volume in different groups. Data are presented as mean ± SEM (*n* = 6/group). *** *p*-value < 0.001 compared with MCAO + placebo-treated group, ^###^ *p*-value 0.001 when compared with naïve control group, ^aaa^ *p*-value < 0.001 when compared with sham + vehicle-treated group.

Figure 3 demonstrated that when compared to the naïve intact control group, rats that received placebo pretreatment and were subjected to sham operation failed to produce the significant change in brain infarcted volume in both the cortex and hippocampus. This information suggested that sham operation and placebo exerted no effect on brain infarcted volume. The current results revealed that MCAO/IR increased brain infarcted volume in both areas just mentioned of the rats that received placebo treatment (*p*-value < 0.001 for all; compared to the sham + placebo treated group). Therefore, the infarcted volume observed in this study should be due to MCAO/IR. Banana peel-derived dietary fiber, banana pulp puree, germinated black Jasmine rice, and piracetam and the developed functional drink (BR) at a dose of 300 mg/kg BW failed to produce a significant decrease in brain infarction volume in the cortex, whereas BR at the doses of 600 and 900 mg/kg BW significantly alleviated an elevation of brain infarction volume induced by MCAO/IR (*p*-value < 0.001 for all; compared to the MCAO + placebo-treated group). Therefore, the reduction in the brain infarcted volume in the cerebral cortex induced by MCAO/IR appeared to be due to the interaction between various components of the developed functional drink. The functional drink at a low dose failed to show that the reduction in brain infarcted volume might be associated with the dose of the tested substance, and various components in the tested substance at this dose were lower than the therapeutic levels, or the improvement in brain infarction might not be high enough to produce a significant change. Interestingly, an elevation of brain infarcted volume in the hippocampus area in rats subjected to MCAO/IR was attenuated by banana pulp puree, germinated black Jasmine rice extract, piracetam, and all doses of BR (*p*-value < 0.001 for all; compared to the MCAO + placebo treated group). These data reflected the vulnerability differences to the tested substances between the cerebral cortex and hippocampus. In this study, it was found that MCAO/IR on the right side produced more brain infarcted volume in the cerebral cortex than in the hippocampus, as shown in Figure 3A,B. The possible explanation for this phenomenon may be associated with the difference in blood supply. The occlusion of the middle cerebral artery affected mainly the cerebral cortex, and the ischemia can also lead to the damage of deeper structures such as the hippocampus due to the branches of this artery, which supply the mentioned area. However, the hippocampus also received blood supply from other blood vessels, such as the posterior cerebral artery and the anterior choroidal artery [33,34].

### 3.2. Changes in Density of the Survival Neurons in Cortex

**Figure 4:**
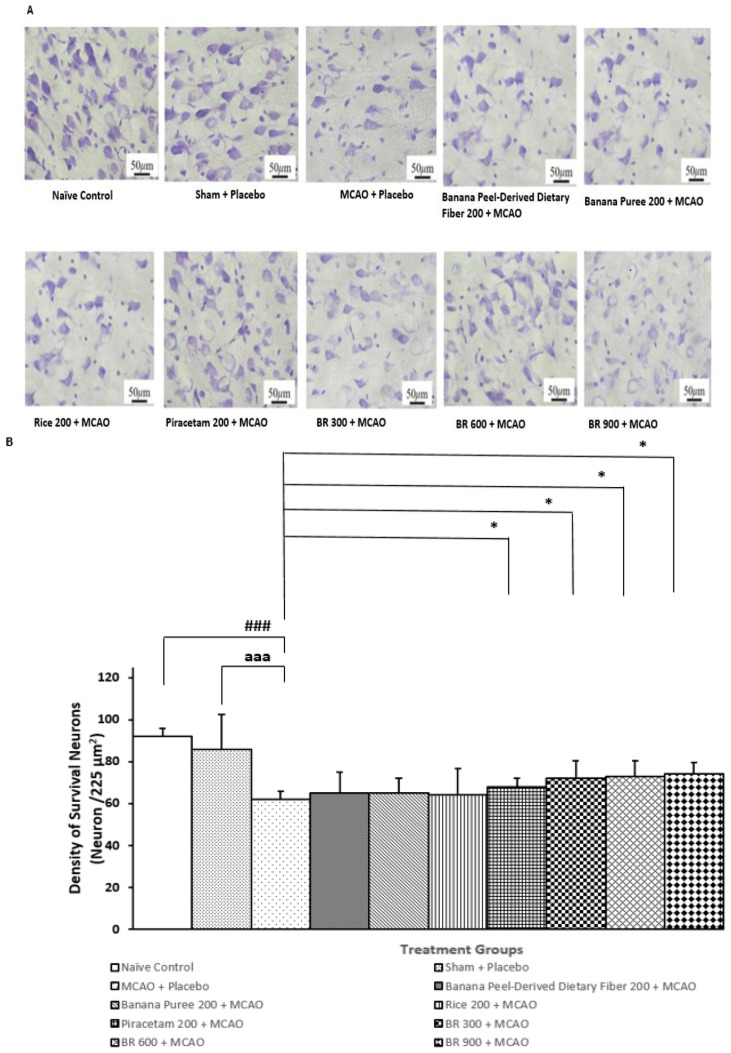
Density of the survival neurons in cortex of various treatment groups. (**A**) Representative photograph of frontal cortex of various treatment groups (**B**) Bar graph of neuron density in frontal cortex of various treatment groups Data are presented as mean ± SEM (*n* = 6/group). * *p*-value < 0.05 compared with MCAO + placebo-treated group, ^###^ *p*-value < 0.001 when compared with naïve control group, ^aaa^ *p*-value < 0.001 when compared with sham + placebo-treated group.

Figure 4 revealed that the sham + placebo treated group failed to produce a significant change in neuron density in the medial frontal cortex (stereotaxic coordinates anteroposterior (AP) 2.5–4.5 mm). This information reflected that the sham operation and vehicle didn’t exert any significant influence on the density of the survival neurons. Rats that were treated with placebo and subjected to MCAO/IR significantly decreased neuron density in this area (*p*-value < 0.001 compared to the Naïve control group; *p*-value < 0.001 compared to the sham + placebo treated group). Therefore, the reduction in neuron density in the cortex was induced by MCAO/IR. This change was mitigated by piracetam, and all doses of BR used in this study (*p*-value < 0.05 for all compared to the MCAO + placebo-treated group). No significant change of this parameter was observed in other treatment groups. These data suggested that the mitigation effects of BR on the reduction in neuron density might be associated with the interactions between various components in BR.

### 3.3. Changes in Inflammatory Markers in Serum

**Figure 5:**
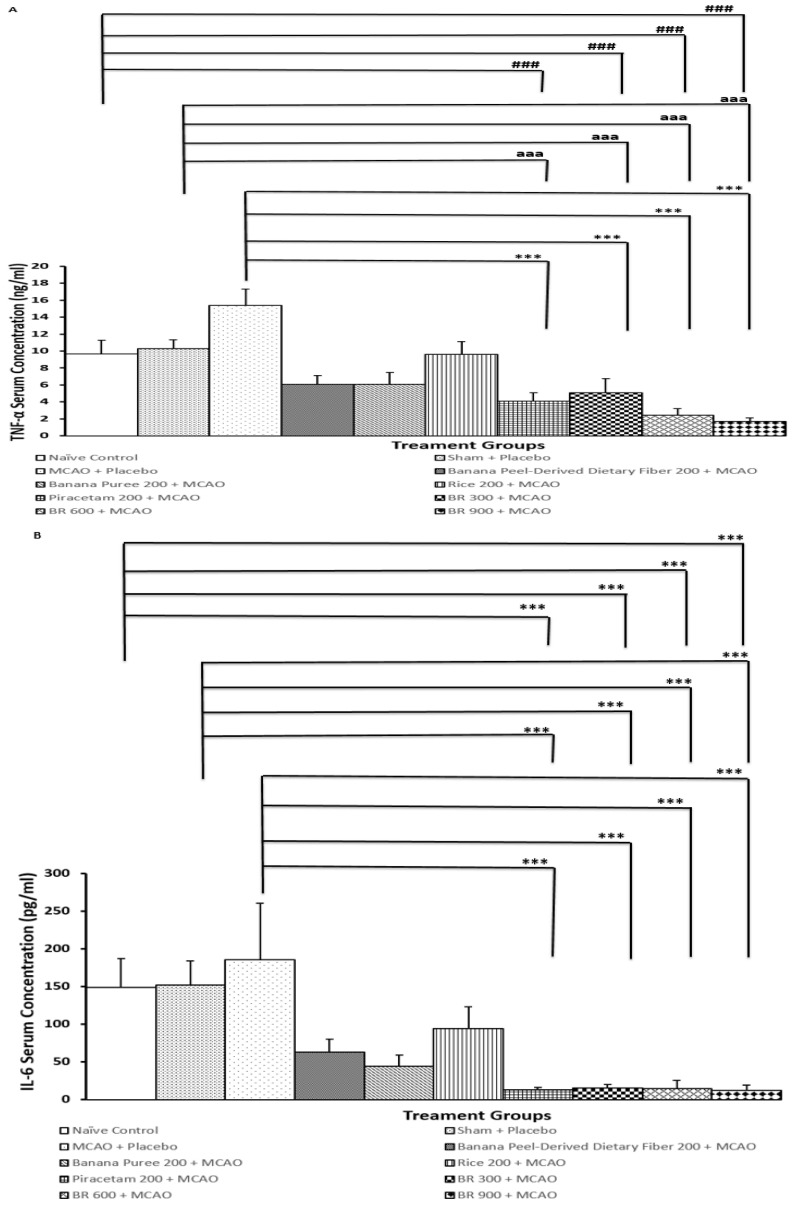
The serum levels of TNF-α and IL-6 of various treatment groups. (**A**) Serum levels of TNF-α (**B**) Serum levels of IL-6. Data are presented as mean ± SEM (*n* = 6/group) *** *p*-value < 0.001 compared with MCAO + placebo-treated group, ^###^ *p*-value < 0.001 when compared with naïve control group, ^aaa^ *p*-value < 0.001 when compared with sham + placebo-treated group.

The inflammatory markers, including serum TNF-α and IL-6, were also determined in order to reflect inflammatory status. Figure 5A,B revealed that rats that were treated with placebo and subjected to sham operation failed to produce significant changes in serum TNF-α and IL6 levels. These findings suggested that sham operation and placebo did not increase serum TNF-α and IL6 levels. Rats that are treated with placebo and subjected to MCAO/IR significantly increased serum TNF-α and IL-6 (*p*-value < 0.001 compared to the naïve intact group; *p*-value < 0.001 compared to the sham + placebo treated group). Therefore, the elevations of both TNF-α and IL-6 might be associated with MCAO/IR. These changes were mitigated by piracetam and BR at the doses of 300, 600, and 900 mg/kg BW (*p*-value < 0.001 for all compared to the MCAO + placebo-treated group). Thus, the mitigation effect of BR on serum TNF-α and IL-6 might be associated with the interaction among various components in BR.

### 3.4. Changes in Oxidative Stress Markers

Table 1 revealed that the sham operation group + placebo-treated group significantly decreased catalase but increased MDA in the cortex (*p*-value < 0.001 for all compared to the naïve control group). Rats that obtained placebo treatment and were subjected to MCAO/IR also showed the reduction in catalase but increased MDA in the area just mentioned (*p*-value < 0.001 for all compared to the naïve control group). Banana peel-derived dietary fiber and banana puree treatments at the doses that were found in the medium dose of functional ingredient in the functional drink mitigated the reduction in this enzyme, but they failed to restore catalase in the cortex of MCAO rats to normal value (*p*-value < 0.01 and 0.001, respectively; compared to the naïve control group). It was found that black rice extract at the dose that presented in the medium dose of the functional ingredient in the functional drink also significantly mitigated the reduction in catalase in the cortex of MCAO rats (*p*-value < 0.01 compared to the naïve control group; *p*-value < 0.01 compared to the placebo + MCAO-treated group; *p*-value < 0.05 compared to the placebo + sham operation-treated group). In addition, this mitigation effect was also observed in MCAO rats that received piracetam and BR at the doses of 300, 600, and 900 mg/kg BW (*p*-value < 0.05, 0.05, 0.01, and 0.001, respectively, compared to the MCAO + placebo-treated group). Although banana peel-derived dietary fiber, banana puree, and rice significantly mitigated the reduction in catalase in cerebral cortex, no significant improvement in MDA was detected in any treatment groups mentioned earlier. This might occur because the increase in catalase induced by the mentioned treatments was not high enough to buffer the increased oxidative stress. However, the significant reduction in MDA was observed in BR treatment at the dosage range used in this study. This phenomenon might be due to the synergistic effect of various components of BR, which in turn enhanced the buffering capacity of other enzymes in addition to catalase or might in turn decrease oxidative stress production.

### 3.5. Assessment of Nrf2 Expression in Cortex

**Figure 6:**
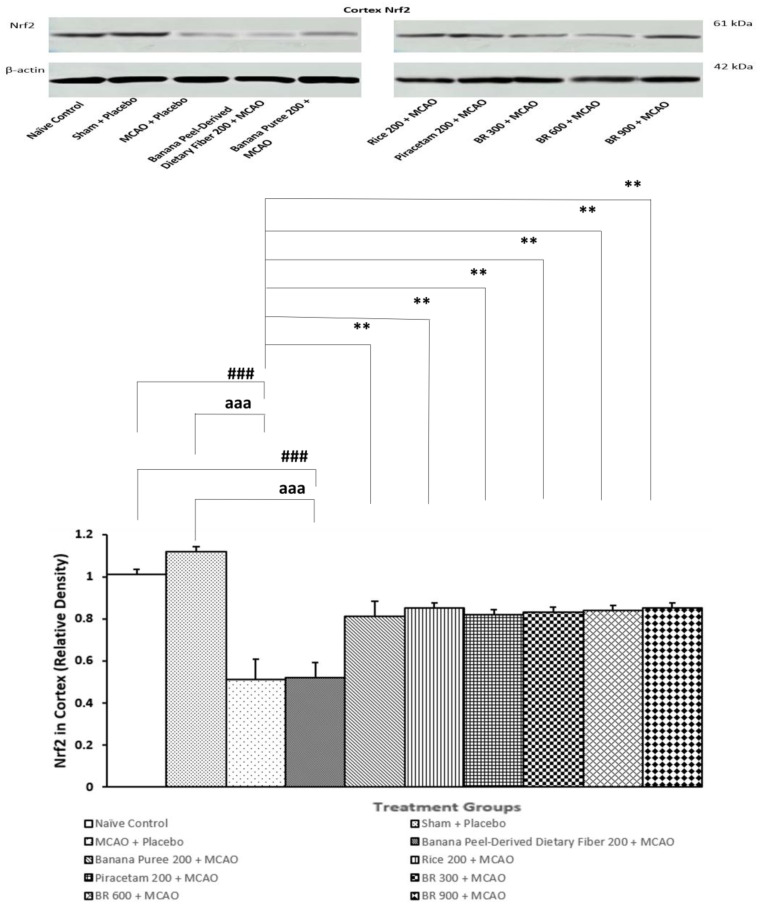
The expression of nuclear factor erythroid 2-related factor 2 (Nrf2) in cortex of various treatment groups. Data are presented as mean ± SEM (*n* = 6/group) ** *p*-value < 0.01 compared with MCAO + placebo-treated group, ^###^ *p*-value < 0.001 when compared with naïve control group, ^aaa^ *p*-value < 0.001 when compared with sham + placebo-treated group.

Figure 6 demonstrated the expression of Nrf2 in the cortex of various treatment groups. Sham + placebo failed to produce a significant change in Nrf2 expression in the cortex, whereas the MCAO + placebo-treated group significantly decreased Nrf2 expression in this area (*p*-value < 0.001 compared to the sham + placebo-treated group; *p*-value < 0.001 compared to the naïve control). These data suggested that sham operation and placebo did not modulate Nrf2 expression in the cortex, while MCAO/IR produced a significant reduction in Nrf2 expression in the mentioned area. However, this change was attenuated by banana pulp puree, germinated black Jasmine rice, piracetam, and all doses of BR (*p*-value < 0.01 for all compared to the MCAO + placebo group. Therefore, the modulation effect of BR in this study might occur partly via banana pulp puree and germinated black Jasmine rice, together with the interactions among various components in BR.

### 3.6. Changes in Lactobacillus and Bifidobacterium *spp.*

**Figure 7:**
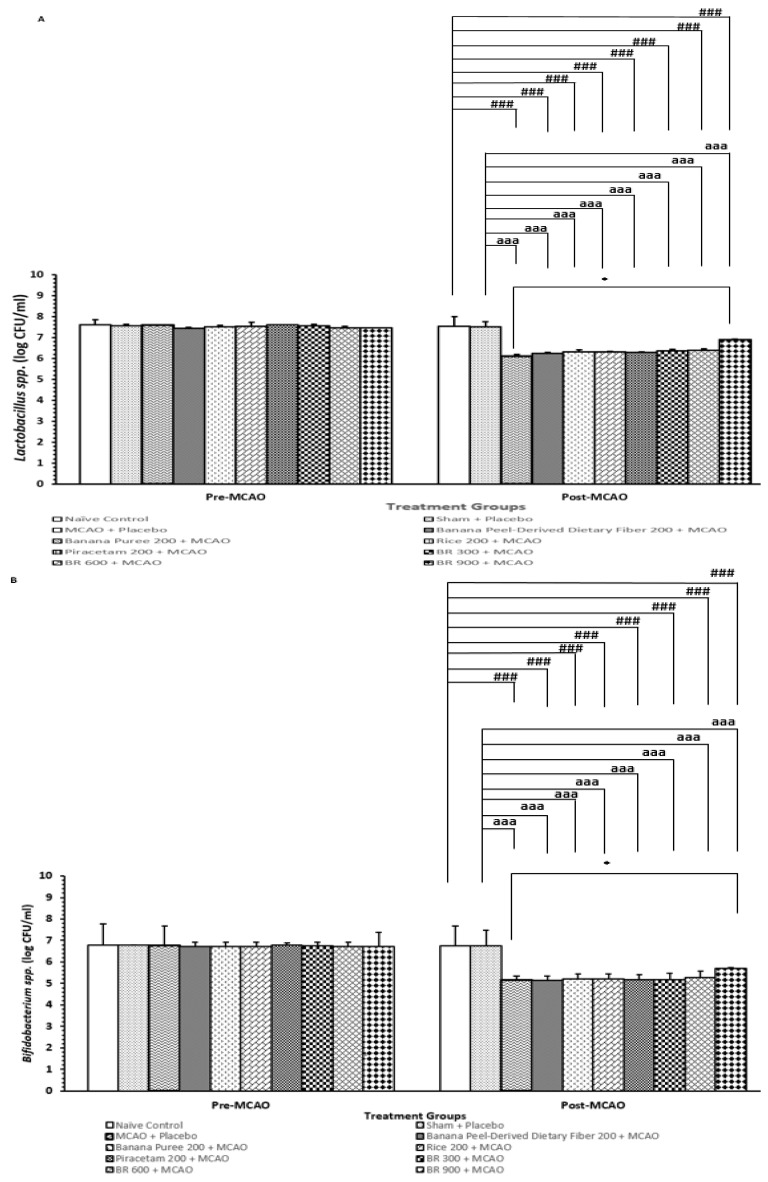
Amount of *Lactobacillus* and *Bifidobacterium* spp. in feces. (**A**) Amount of *Lactobacillus* spp. in feces. (**B**) Amount of *Bifidobacterium* spp. in feces. Data are presented as mean ± SEM (*n* = 6/group) * *p*-value < 0.05 compared with MCAO + placebo-treated group, ^###^ *p*-value < 0.001 when compared with naïve control group, ^aaa^ *p*-value < 0.001 when compared with sham + placebo-treated group.

Figure 7A,B demonstrated that rats that obtained a placebo and were subjected to a sham operation significantly decreased the amount of *Lactobacillus* and *Bifidobacterium* spp. (*p*-value < 0.001 for all compared to the sham + placebo treated group). These reductions of both bacteria spp. were attenuated by a high dose of the developed functional drink (*p*-value < 0.05 for all compared to the MCAO + placebo-treated group). No significant change of this parameter was observed in other groups. The modulation effect of BR on the density of both bacteria spp appeared to be associated with the interaction among various components in BR.

## 4. Discussion

The current data reveals that rats treated with the functional drink containing the functional ingredients from banana and black Jasmine rice show a significant reduction in brain infarcted volume, MDA, and inflammatory cytokines such as TNF-α and IL-6 levels, together with an increase in neuron density, Nrf2, and catalase in the cortex. The reductions of serum TNF-α and IL-6 are also detected. Moreover, the elevation of both *Lactobacillus* and *Bifidobacterium* spp. in the feces of rats that received the high dose of the functional drink is also present.

Recently, it has been demonstrated that Nrf2 plays an important role in the maintenance of an anti-inflammatory environment and optimal cellular function for T cells, thereby reducing tissue oxidative stress and inflammatory damage [35]. It has been demonstrated that Nrf2 suppresses the inflammatory response by suppressing TNF-α [36] and IL-6 [37]. In addition, Nrf2 can also directly increase the expression of antioxidant enzymes, which in turn decreases oxidation products such as MDA, a lipid peroxidation product, and alleviates brain damage induced by oxidative stress in ischemic stroke [38,39]. The current data reveal the close relationship between brain infarction and changes in the biomarkers of oxidative stress status and inflammation. Based on these pieces of information, the developed function drink at the dosage range used in this study may increase Nrf2, giving rise to the reduction in oxidative stress and inflammation. Although brain degeneration shows an association with brain infarction, our data fail to show the close relationship between these 2 parameters. The explanation of this finding may be attributed partly to the method of detection. Brain infarction assessment with the method used in this study relied on the oxidation of TTC by intact mitochondrial dehydrogenase, which yields the carmine-red product, formazan. The degenerated cell lacks this enzyme, so it will be presented as white-colored tissue [40]. However, the detection of survival cells in the cortex detects only the cells with dense Nissl substance. Therefore, the degenerating cell without the clear presentation of chromatolysis will not be counted and gives rise to a lesser magnitude of change in neuron density when compared to brain infarction volume. The increase in neuron density in the cortex also reflects an improvement in the trophic effect of the developed functional drink.

The high dose of the developed functional drink also increases the amount of *Lactobacillus* and *Bifidobacterium* spp. Based on the information that *Bifidobacterium* spp. and *Lactobacillus* spp. improve inflammation in stroke [41,42], they may also contribute to the role of the improvements of brain damage and brain edema by modulating the gut–brain–axis. Further research is essential to confirm this positive modulation effect and to provide a better understanding regarding detailed mechanisms.

Taking all data together, the improvement in brain damage observed in this study may occur partly via the improvements in oxidative stress status and inflammation. The developed functional drink may increase Nrf2 expression, which in turn increases antioxidant enzymes such as CAT, giving rise to a reduction in oxidative stress and MDA level. An elevation of Nrf2 also decreases inflammatory cytokines such as TNF-α and IL-6, leading to the improvement of brain damage. In addition, the modulation of gut microbiota, which in turn exerts a positive modulation effect on gut–brain–axis also plays a role, particularly at high dose levels of the developed functional drink.

The current results have revealed that all doses of the functional drink improve neuronal density in the cortex, whereas the banana peel-derived dietary fiber alone, banana pulp puree alone, and black Jasmine rice extract alone produce significant changes in some parameters just mentioned. Therefore, this information points out that the positive modulation effects of the developed functional drink containing the functional ingredient derived from banana and black jasmine rice in this study appear to be associated with the interaction of various ingredients in the mixture that served as the functional ingredient, including dietary fiber and puree from banana and extract from germinated black jasmine rice. Moreover, our data also support the optimized benefit of cerebro-protection induced by the interaction among various ingredients of this novel functional drink. This phenomenon also contributes to the lack of dose–dependent response manner of the developed functional drink.

The limitation of this study is the lack of information that links the increase in microbiota such as *Lactobacillus* and *Bifidobacterium* spp. induced by the consumption of the developed functional drink and the gut–brain–axis stimulation and the neuroprotection. Therefore, further research to elucidate these points is required to provide a better understanding about the detailed mechanisms.

## 5. Conclusions

The current results show that when compared to MCAO/IR rats that received a placebo, MCAO/IR rats that were treated with the functional drink containing the functional ingredients from banana and black Jasmine rice (BR) show a significant reduction in brain infarcted volume, MDA, and inflammatory cytokines such as TNF-α and IL-6 levels, together with an increase in neuron density, Nrf2, and catalase in the cortex. The reductions of serum TNF-α and IL-6 are also detected. Moreover, the elevation of both *Lactobacillus* and *Bifidobacterium* spp. in the feces of rats that received the high dose of the functional drink is also present. Owing to the mentioned information, the developed functional drink from banana and black jasmine rice has the potential to reduce the hazard from stroke attacks. The functional ingredient obtained from the mixture of banana peel-dietary fiber, banana pulp, puree, and germinated black jasmine rice extract provides a better benefit than the treatment with each ingredient alone due to the positive interaction effect. The possible underlying mechanism may occur via multi-pathways, including an increase in Nrf2, which in turn improves oxidative stress, inflammation, and cerebral blood flow, and shows the positive modulation effect on gut microbiota such as *Lactobacillus* and *Bifidobacterium* spp., which in turn may improve the gut–brain–axis. The detailed mechanisms require further exploration. Our findings suggest that the novel functional drink may be the potential functional drink for protecting against ischemic stroke. However, a clinical trial is essential to confirm this positive modulation effect.

## Figures and Tables

**Table 1:** Oxidative stress markers, including malondialdehyde (MDA) level and catalase (CAT) activity, in cortex of various treatment groups. Data are presented as mean ± SEM (*n* = 6/group) *, **, *** *p*-value < 0.05, 0.01, and 0.001, respectively; compared with MCAO + placebo-treated group, ^#^, ^##^, ^###^ *p*-value < 0.05, 0.01, and 0.001, respectively, when compared with naïve control group, ^a^, ^aaa^ *p*-value < 0.05 and 0.001, respectively, when compared with sham + placebo-treated group.

Groups	Cortex MDA (μmol/L)	Cortex Catalase (kU/L)
Naïve Control	5.31 ± 1.1 ^a,^**	217. 99 ± 10.17 ^aaa,^***
Sham + Placebo	12.71 ± 0.25 ^###^	147.65 ± 1.16 ^###^
MCAO + Placebo	14.83 ± 0.33 ^###^	145.50 ± 1.41 ^###^
Banana Peel-Derived Dietary Fiber 200 + MCAO	10.15 ± 1.97	155.41 ± 1.36 ^##^
Rice 200 + MCAO	10.55 ± 1.76	183.04 ± 1.84 ^##,a,^**
Banana Puree 200 + MCAO	10.64 ± 1.63	165.84 ± 4.84 ^###^
Piracetam 200 + MCAO	7.16 ± 0.15 *	178.16 ± 5.61 ^##,^*^,a^
BR 300 + MCAO	7.61 ± 0.31 *	173.84 ± 6.10 ^##,^*^,a^
BR 600 + MCAO	6.94 ± 0.37 *	177.55 ± 16.03 ^#,a,^**
BR 900 + MCAO	5.42 ± 0.78 **	194.73 ± 10.34 ^aaa,^***

## Data Availability

The data presented in this study are available on request from the corresponding author. The data are not publicly available due to a trade secret, and petty patent registration process.
